# Effect of exercise intensity on weight changes and sexual hormones (androstenedione and free testosterone) in female rats with estradiol valerate-induced PCOS

**DOI:** 10.1186/1757-2215-7-37

**Published:** 2014-04-08

**Authors:** Maryamosadat Miri, Hojatolah Karimi Jashni, Farzaneh Alipour

**Affiliations:** 1 Exercise Physiology, Jahrom University of Medical Sciences, Jahrom, Fars, Iran; 2 Department of Anatomy, Jahrom University of Medical Sciences, Jahrom, Iran; 3 Student Research Committee, Jahrom University of Medical Sciences, Jahrom, Fars, Iran

**Keywords:** Exercise intensity, Weight change, Androstenedione, Free testosterone

## Abstract

**Introduction:**

Weight gain and fat accumulation are predisposing factors of PCOS. Life-style modification, including increasing physical activity, is the first line approach in managing PCOS. The objective of this study is to assess the effect of exercise intensity on weight changes, androstenedione and free testosterone level in female rats with estradiol valerate induced PCOS.

**Method and materials:**

40 female Wistar rats were selected (180 ± 20 g). They had every 2 to 3 consecutive estrous cycles during 12 to 14 days. The study was approved by ethical committee of Jahrom University of Medical Sciences. The first two groups were divided into control (n = 10) and polycystic (n = 30) that were induced PCOS by estradiol valerate injection after 60 days. The polycystic groups were divided into three groups of sham (n = 10), experiment group with low-intensity exercise (pco + l.exe) (n = 10) and experiment group with moderate intensity exercise (pco + m.exe) (n = 10). Exercises were performed during 6 sessions of 60 minutes per week for 8 weeks. (Moderate intensity: 28 m/min-70%–75%VO2Max. Low intensity (20 m/min-50%–55%VO2Max) running at 0 slope, 1 h/day, 6 days/week). ANOVA and LSD test were used for data analysis.

**Results:**

In the present study, no significant differences were found in the decrease of total weights of rats. And also androstenedione level changes in experiment groups were higher compared to control group but no significant differences were found, also free testosterone level was significantly higher than the observer group.

**Conclusion:**

According to weight changes and sexual hormones (Free testosterone and androstenedione) exercise training especially with low intensity may improve symptoms of polycystic ovary syndrome.

## Introduction

It is certain that infertility is one of the main problems in today’s medicine and its rate is increasing from 1955 and 10%–15% of the couples are suffering from that [1]. One of the causes of infertility is polycystic ovary syndrome (PCOS). PCOS is the most common endocrine abnormality in premenopausal women. It was first described by Stein and Leventhal in 1935, who found an association between amenorrhea, hirsutism, and obesity with polycystic ovaries. The authors reported on bilaterally enlarged ovaries, with a thick and whitened capsule [2]. This syndrome is characterized by hyperandrogenism, ovulatory dysfunction, irregular menstrual cycles, imbalance of sex hormones and polycystic ovarian morphology. Metabolic disturbances, such as insulin resistance and obesity are also associated with PCOS. It is thought to have a genetic etiology behind this syndrome, the severity and course of the disease is determined by lifestyle changes, especially body mass index [3].

On the other hand, the importance of exercise and mental health of individuals and society is obvious and is inseparable from the health of body and spirit. Attention to women's exercise as much of their bodies’ physiological needs is essential [4]. Physical activity and exercise cause levels of some hormones increase or decrease compared to resting level. Physical activity reduces estrogen and steroid hormone production [5]. Lifestyle intervention studies incorporating increased physical activity with reduced caloric intake show an improvement in ovulatory function, circulating androgen levels, inflammatory pattern, and insulin sensitivity in women with PCOS [6]. Furthermore, certain single-nucleotide polymorphisms associated with obesity contribute to elevated body mass index (BMI) in PCOS, supporting the concept that its phenotypes are a consequence of a polygenic mechanism [7].

Controversy exists about the effect of obesity on serum androgen production in PCOS. Some investigators have reported that testosterone and androstenedione levels are similar in obese and non obese PCOS patients [8,9]. However, it is well known that obesity generates a decrease in the sexual hormone-binding globulin, and therefore an increase in the levels of free androgens [10,11]. In contrast, dynamic studies have shown lower androstenedione levels in obese PCOS patients than in non obese PCOS patients [12,13]. Researchers believe that regular and light sports are a safe method. The effects of aerobic exercise on polycystic ovary syndrome was assessed in some papers, it is stated that, apart from the changes in body fat, level of sex hormones have been changed [6].

In the past, the effects of regular exercise with high intensity (80–85% maximal oxygen uptake), moderate intensity (70–75% maximal oxygen uptake) and low intensity (50–55% maximal oxygen uptake) were assessed. The result showed testosterone associated with high-intensity group was lower than inactive control group [14]. Since, the effect of exercise intensity in PCOS have not been assessed and regarding the importance of physical activity in rehabilitation of hormonal imbalances, we conducted this study to evaluate the effect of exercise intensity on weight changes, androstenedione and free testosterone levels in female rats with estradiol valerate-induced PCOS.

## Method and materials

### Animals

40 female Wistar rats were selected (180 ± 20 g). They had every 2 to 3 consecutive estrous cycles during 12 to 14 day. The rats were selected from Shiraz University of medical sciences and were kept in animal house of Jahrom University of medical sciences. Their cages were disinfected 3 times a week with alcohol 70% and enough water and also appropriate bottle was provided for them.

### Approval

The study was approved by ethical committee of Jahrom University of Medical Sciences and morality was regarded.

### Induction of PCOS

PCO phenotype is induced by of a variety of hormonal and non-hormonal methods, including testosterone, estradiol valerate, and dehydroepiandrosterone (DHT), adrenocorticotropic and long-term use of light. In this study, we used estradiol Valerate as an inducer. 30 rats were selected randomly from 40 ones. 4 mg estradiol validate which was dissolved in 0.2 mg Sesame oil was injected (IM) in their thigh.

### Design

The first two groups were divided into control (n = 10) and polycystic (n = 30) that were induced PCOS by estradiol valerate injection after 60 days. The polycystic groups were divided into three groups of sham (n = 10), experiment groups (PCOS plus low-intensity exercise (n =10) and experiment group (PCOS plus moderate intensity exercise (n =10)). Exercises were performed during 6 sessions of 60 minutes per week which lasted 8 weeks. Moderate intensity: (28 m/min-70%–75%VO2Max. Low intensity (20 m/min-50%–55%VO2Max)running at 0 slope, 1 h/day, 6 days/week. The mice were anesthetized, then, of 5 ml of blood was obtained directly from the heart and was used for ELIZA. Exercise Programs.

### Exercise training protocol

The training group was exercised on a rodent motor-driven treadmill at a 0° slope for 60 min/day, 6 days/wk for 8 wk. During the 1st wk of training the rats ran at treadmill speed of 10 m/min for 15 min for adaptation. During the 2nd and 3rd wk of training the treadmill speed and exercise duration increased step by step until the animals ran for 60 min/day. The treadmill speed and exercise duration were then held constant for the remainder of the training period. We kept training frequency (6d/wk) and duration (60 min/d) constant. The exercise plan started with short-duration and light movements and gradually increased in intensity.

### Vaginal smears and blood sampling

The estrus cycle stage was determined by microscopic analysis of the predominant cell types obtained via the vaginal smears taken daily (24) Vaginal smear test was taken within 60 days for reassurance of induction of PCOS in experiment groups. After vaginal smear test, we used animals having 2 or 3 regular estrous cycle within 12–14 days. Blood sampling directly from their heart was done through a 5 cc syringe after 32 hours following the last session. After isolation of blood serum, concentration of free testosterones, androstenedione was measured by ELIZA in Jahrom University of Medical Sciences.

### Blood collection and tissue preparation

To derogate the effect of acute exercise, the rats were eventually anesthetized with diethyl ether and sodium pentobarbital (50 mg/kg, intraperitioneal injection) after a 12-h fast and 32 h after the last training session and their blood was gleaned from the abdominal aorta. Tubes containing plasma sample aliquots were kept frozen at -80°C until being analyzed.

### Measurement

BioVendor kit was used for measurement of Free testosterone and androstenedione level in ELISA test.

### Analyzing method

ANOVA test was used for comparison of mean and Standard deviation of hormones and tukey test for multi comparison of different groups was used in the studying groups (p < 0.05) was considered as significant difference. ANOVA and LSD test for normal distributions was used.

## Results

### Results of final weights of rats

**Figure 1:**
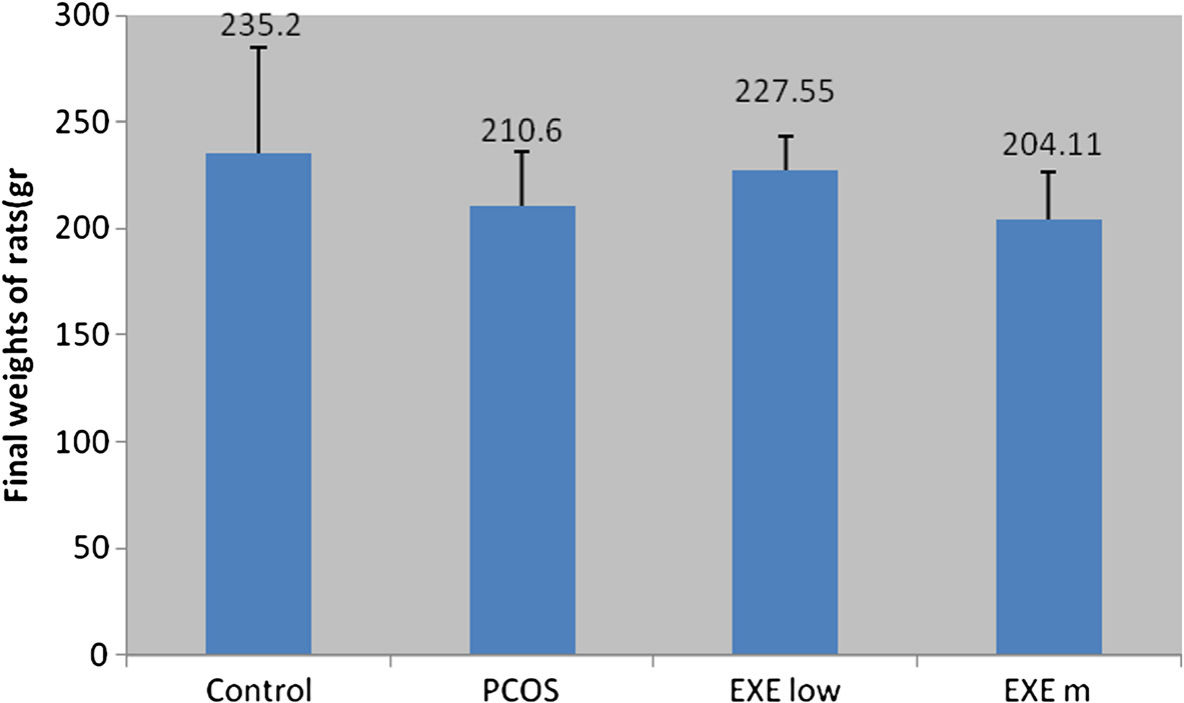
Changes of final weights of rats in experiment groups.

The results show that, no significant differences were found in the final weights of the rats compared to the control group. Final weights of rats in pco + exe.l and pco + exe.m did not have significant differences comparing the poly cystic group and also weight loss in pco + exe.l group was not significant comparing pco + exe.m group. Variance analysis of data was not significant. Also final weights of the rats have normal distribution. Regarding fissure value and data analysis, this factor does not have significant differences (Tables 1 and 2, Figure 1).

**Table 1:** Comparison of means of all variables

	**Moderate intensity**	**Low intensity**	**PCOS**	**Control**
Free testosterone(ng/ml)	0.494 ± 0.26 *	0.319 ± 0.12	0.178 ± 0.08	0.343 ± 0.26
Androstenedione (ng**/**ml)	0.347 ± 0.172	0.273 ± 0.09	0.278 ± 0.16	0.261 ± 0.09
Weight total (gr)	28. 62 ± 17.70	34.55 ± 6.22	32.8 ± 19.27	28.66 ± 19.20
Final weight (gr)	204.11 ± 22.89	227.55 ± 16.14	210.6 ± 25.47	235.2 ± 49/82

* significant difference comparing PCOS (p < 0.05).

**Table 2:** Variance analysis of final weights of rats (gr)

**S.V**	**F.S**	**M.S**	**S.S**	**D.F**
Group	1.964 ns	1990.76	5972/28	3
Error	-	1013.62	34463/11	34
Total	-	-	40435/39	37

ns (non-significant).

### Results of total body weight of rats

**Figure 2:**
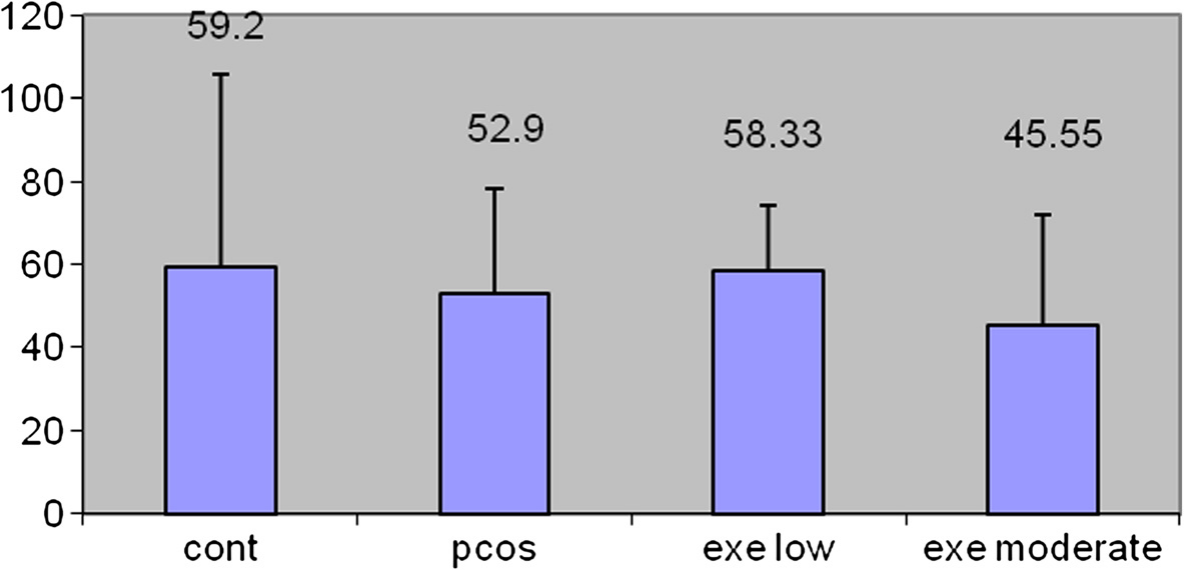
Changes of total weights of rats in experiment groups.

Total weight means the difference of primary and final weights of rats. Although weight reduction is seen in our results, no significant difference was found in the total weights in the experiment groups comparing control group. And also data of the pco + exe.l group did not have significant differences comparing the polycystic group. No significant difference was found according to variance analysis (Table 3, Figure 2).

**Table 3:** Variance analysis of total weights of rats (gr)

**S.V**	**F.S**	**M.S**	**S.S**	**D.F**
**Group**	**0.282 ns**	68/49	205/48	3
Error	-	243/09	5834/23	24
**Total**	**-**	**-**	**6039/71**	**27**

ns (non-significant).

### Results of body weight in 2 month protocol

**Figure 3:**
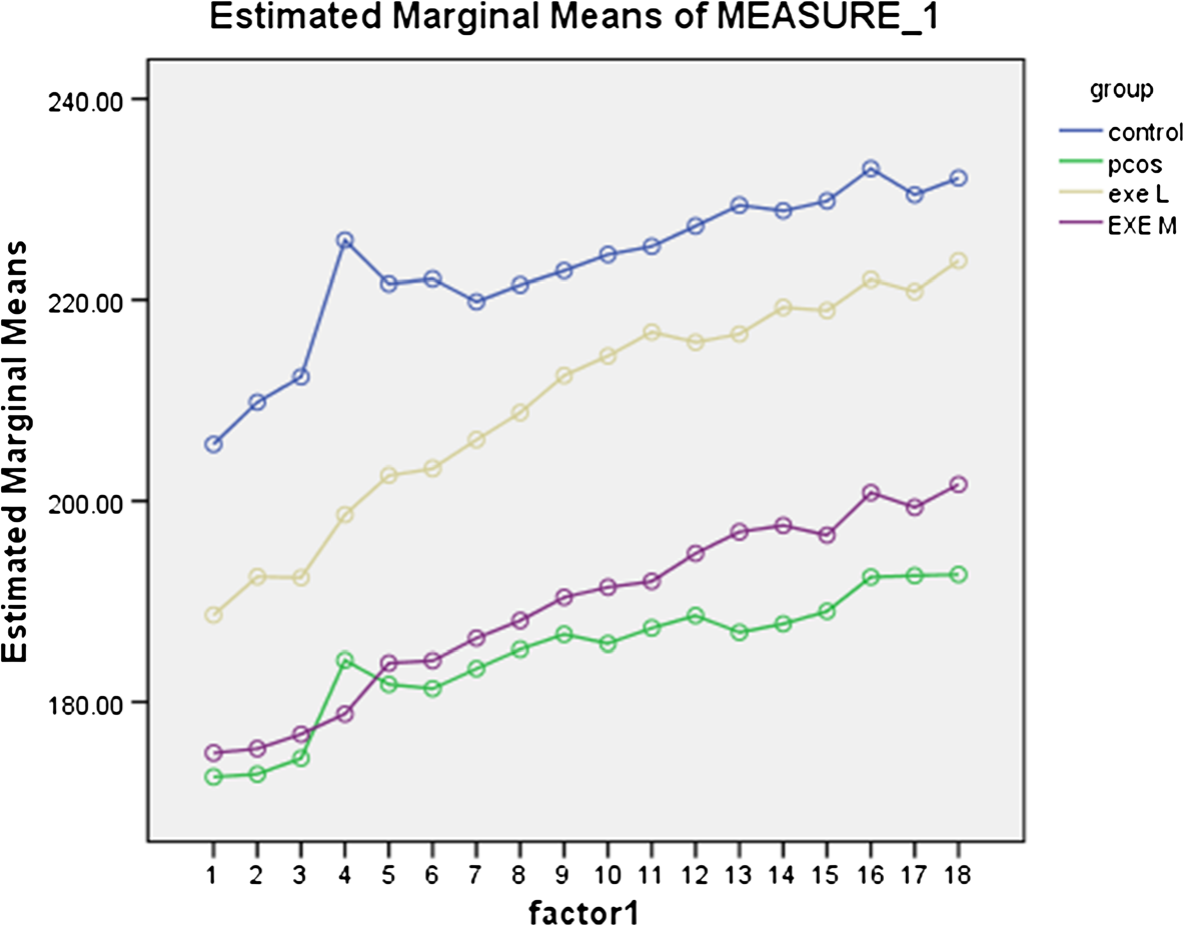
Weight changes during 2 month protocole.

Total body weight was measured within 2 month and recorded 18 times. Since most of weight changes were significant, data which were not significant are reported. Both experiment and polycystic groups did not have significant differences comparing the control group in this protocol. Weight changes are reported as below (Figure 3):

Weight of 6th time is not significant compared to the 5th and 4th ones. Also 10th compared to 9th and 15th compared to 12th, 13th and 14th ones furthermore, 18th compared to 16th were not significant.

### Results of free testosterone changes in experiment group

**Figure 4:**
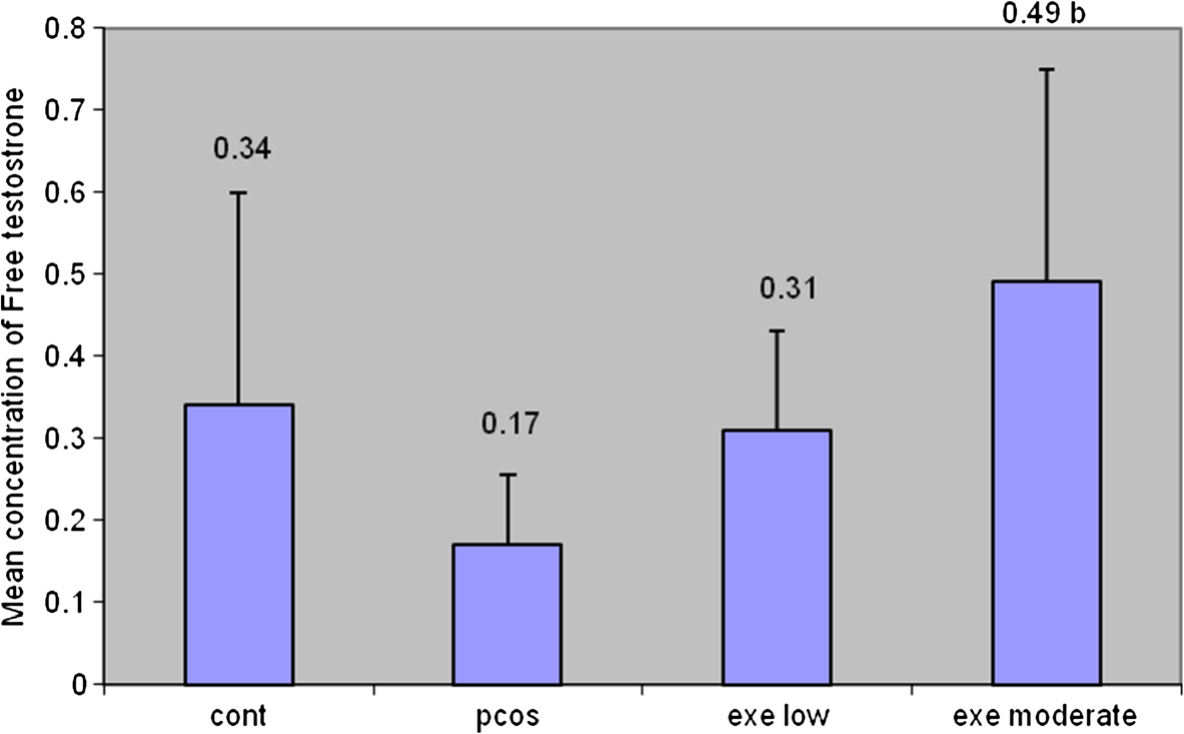
Changes of Free testosterone in experiment groups.

The PCO + exe.m group showed a significant rise in free testosterone. No significant differences were found in the pco + exe.l group and polycystic group comparing the control group. Also pco + exe.l group did not have significant difference compared to polycystic group. The diagram shows that it has normal distribution. Also variance analysis was significant (Table 4, Figure 4).

**Table 4:** Variance analysis of free testosterone (ng)

**S.V**	**F.S**	**M.S**	**S.S**	**D.F**
Group	0. 015*	0.159	0.476	3
Error	**-**	0.040	1.353	34
**Total**	-	-	1.829	37

* (significant) p < 0.05.

### Results of androstenedione changes in experiment group

**Figure 5:**
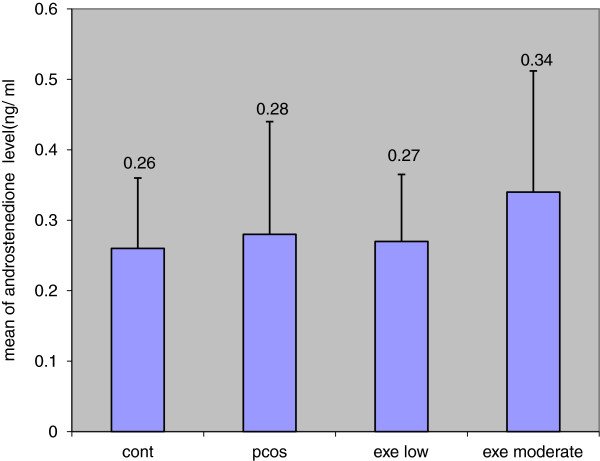
Changes of androstenedione in experiment groups.

Results showed that, androstenedione hormone changes in experiment group did not have significant difference compared to control group. There was no statistically significant difference between the decrease in androstenedione concentration in PCO + exe.l compared to Sham and there was no statistically significant difference between the increase in androstenedione concentration in pco + exe.m compared to sham and also the difference was not significant in PCO + exe.l group compared to PCO + exe.m. Androstenedione changes have normal distribution considering the diagram (Table 5, Figure 5).

**Table 5:** Variance analysis of androstenedione hormone (ng/ml)

**S.V**	**F.S**	**M.S**	**S.S**	**D.F**
Group	0. 720 ns	0.014	0.041	3
Error	**-**	0.019	0.649	34
Total	**-**	-	0.690	37

ns (non-significant).

## Discussion

This study demonstrated that low intensity exercise may cause weight reduction and modify sexual hormones(androstenedione and Free testosterone) in polycystic ovary syndrome after 8 week. Polycystic ovary syndrome (PCOS) is related to the chronic anovulation, hyperandrogenemia, insulin resistance (IR)/hyperinsulinemia, and a high incidence of obesity.

It is emphasized that the most preferred and most effective method of treatment for PCOS is lifestyle modification. Researchers believe that PCOS is accompanied with androgen hormone rise and obesity. Although weight loss improves practically every parameter of PCOS, Wright et al. [15] concluded that differences in dietary intake and physical activity alone are not sufficient to explain differences in weight between women with and without PCOS. Our study confirms the findings by Wright et al. about lifestyle changes [9].

No significant differences were found in the final weights of rats in PCO + exe.l compared to the PCO + exe.m group and also the difference was not statistically significant between the total weights of rats in PCO + exe.m compared to PCO + exe.l group. Their weights were measured within 2 month and weight changes were reported daily. Weight changes were significant in 2nd up to 18th days comparing the first day. Some papers indicated that Physical activity has been found lower in PCOS patients than in control women [15]. The changes in lifestyle that incorporate an increase of physical activity and limited caloric intake have been beneficial in some studies. Regular physical activity is an important component to support the long-term reduction of overweight; however, the results are minimal with exercise alone [9]. An increase in physical activity is recommended for women with obesity and PCOS, as long as cardiovascular and orthopedic limitations are taken into consideration [16]. These are in consistent with our results.

Free testosterone rise in PCO + exe.m was significant comparing Sham. Free testosterone rise could be due to obesity and binding globulin reduction [17,18]. Effect of exercise intensity and its duration on menstruation have not been monitored. Another research showed that, significant rise of free testosterone was observed in women exercising with 75% intensity. They indicated that, rise of hormone is because of decrease in clearance of testosterone due to hepatic blood serum flow reduction [19]. Other studies have found that obesity generates an increase of testosterone levels in PCOS patients [20-22]. But in our study, free testosterone changes in PCO + exe.l shows the sufficiency of low exercise intensity.

Although there are some researches over exercise effect on PCOS, there was not done over the exercise intensity effects on weight changes and sexual hormones (androstenedione and Free testosterone)in PCOS. Hence the present study demonstrated that low exercise intensity may modify weight changes and sexual hormones (androstenedione and Free testosterone) in polycystic ovary syndrome after 8 week better than moderate intensity.

## Conclusion

Here we provided evidence that both low and moderate exercise intensity might enhance polycystic ovary syndrome and decrease its complications due to its effect on weight reduction and sex hormones (androstenedione and Free testosterone). Based on our results, low intensity exercise might be more effective and improve its symptoms.

## Competing interests

The authors have no conflict of financial interests.

## Authors’ contributions

MM and HK evaluated the sexual hormone analysis.MM and FA performed the statistical analyses and drafted the manuscript. MM organized the exercise training and set the protocol, set the weight change and did the vaginal smear test.MM and FA performed ELISA test.HK helped draft the manuscript.MM and FA translated the manuscript and prepared it. All authors read and approved the final manuscript.
